# Occlusion of two semicircular canals does not disrupt normal hearing in adult mice

**DOI:** 10.3389/fneur.2022.997367

**Published:** 2022-09-15

**Authors:** Tianying Wang, Huizhan Liu, David Z. He, Yi Li

**Affiliations:** ^1^Department of Otorhinolaryngology-Head and Neck Surgery, Beijing Tongren Hospital, Capital Medical University, Beijing, China; ^2^Department of Biomedical Sciences, Creighton University School of Medicine, Omaha, NE, United States

**Keywords:** semicircular canal occlusion, vertigo, auditory function, endocochlear potential, mice

## Abstract

Vertigo is a debilitating disease affecting 15–20% of adults worldwide. Vestibular peripheral vertigo is the most common cause of vertigo, often due to Meniere's disease and benign paroxysmal positional vertigo. Although some vertigo symptoms can be controlled by conservative treatment and/or vestibular rehabilitation therapy, these treatments do not work for some patients. Semicircular canal occlusion surgery has proven to be very effective for these patients with intractable vertigo. However, its application is limited due to concern that the procedure will disrupt normal hearing. In this study, we investigated if occlusion of two semicircular canals would jeopardize auditory function by comparing auditory function and hair cell morphology between the surgical and contralateral ears before and after the surgery in a mouse model. By measuring the auditory brainstem response and distortion product otoacoustic emission 4 weeks post-surgery, we show that auditory function does not significantly change between the surgical and contralateral ears. In addition, confocal imaging has shown no hair cell loss in the cochlear and vestibular sensory epithelia, and scanning electron microscopy also indicates normal stereocilia morphology in the surgical ear. More importantly, the endocochlear potential measured from the surgical ear is not significantly different than that seen in the contralateral ear. Our study suggests that occlusion of two semicircular canals does not disrupt normal hearing in the mouse model, providing a basis to extend the procedure to patients, even those with normal hearing, benefitting more patients with intractable vertigo attacks.

## Introduction

Vertigo has a high incidence rate worldwide, affecting between 15 and 20% of adults each year. Its prevalence rises with age, especially in adults over the age of 60. Long-term vertigo causes mood changes and leads to other serious consequences, such as falls, varying degrees of disability, and accidental deaths (1, 2).

Vestibular peripheral vertigo is the most common cause of vertigo, accounting for 71% of all vertigo patients (2, 3). Among them, Meniere's disease accounts for ~1.0–1.6%, and 34% of vertigo patients with benign paroxysmal positional vertigo. These patients often require vestibular rehabilitation therapy and/or other conservative treatment to control vertigo symptoms (4–6). However, these treatments are often ineffective for some patients with intractable vertigo attacks.

Semicircular canal occlusion (SCO) is considered to be an effective treatment of vertigo in patients who are not responsive to conservative treatments. SCO is a surgical operation in which the endolymphatic flow is blocked by bone wax, bone shavings, fascia, biological glue or a laser after drilling in the bony wall of the semicircular canal (7, 8). At present, posterior semicircular canal occlusion (PSCO) is most commonly used in the treatment of benign paroxysmal positional vertigo, and its curative effect has been affirmed. In recent years, PSCO, combined with lateral semicircular canal occlusion (LSCO) and three semicircular canals occlusion, have also been used in treatment of Meniere's disease, and its effectiveness has been recognized (9). However, SCO has also been reported to cause hearing loss in some patients, while no hearing loss has been observed in other patients (9, 10). The controversy surrounding SCO's potential side effects on auditory function has limited the treatment's availability to patients with intractable vertigo attacks, and have moderate to severe hearing loss (9–13). Therefore, determining whether SCO is safe for maintaining auditory function is essential to further expand SCO's availability for patients with other forms of vertigo, such as vestibular peripheral vertigo.

The goal of our study is to investigate if SCO would disrupt the auditory function. We examined auditory function, hair cell morphology, and endocochlear potential (EP) in a mouse model to determine if normal hearing is still retained after a surgical procedure that blocks the two semicircular canals (i.e., combination of PSCO and LSCO). If the SCO surgery is observed to have a minimal impact on the auditory function of animal models, our experiments will provide a stronger basis for extending the treatment to patients with normal hearing, benefitting a wider range of patients suffering from intractable vertigo attacks.

## Materials and methods

### Animals

C57BL/6 mice with both sexes at 4 weeks of age were used for experiments. The care and usage of mice were approved by the Animal Ethics Review Committee of Beijing Capital Medical University.

### Surgery

Animals were anesthetized through an intraperitoneal injection of ketamine HCl (120 mg/kg) and xylazine HCl (7 mg/kg). After anesthesia administration, the animal was placed on a preheated pad with a preset temperature of 37°C. The canalostomy procedure, including locating and exposing the posterior semicircular canal (PSC) and lateral semicircular canal (LSC) in neonatal and adult mice, has been previously described in detail (14). The surgery was performed only on one ear. Two small holes were made on PSC and LSC, respectively, using a miniature electric drill. After the small hole was drilled in the bony wall of LSC and PSC, translucent tissue and endolymph leakage were observed. The two holes were then blocked with a piece of muscle tissue and surgical bone wax. The left or right ear was randomly selected as the surgical ear and the contralateral ear was used as control.

### Measurement of auditory brainstem response and distortion product otoacoustic emission

ABRs were used to determine hearing threshold. ABRs were recorded using a Tucker-Davis Technologies workstation with SigGen32 software (Tucker-Davis Technologies Inc., Alachua, FL, USA) in a sound-isolated chamber as previously described (15). Mice were anesthetized and placed on a temperature-controlled heating pad. Tone pips with frequencies of 4, 8, 12, 16, 22, 32, 40, and 50 kHz were delivered to the ear canal with an EC1 electrostatic speaker (Tucker-Davis Technologies, Alachua, FL, USA). ABR signals were collected with subcutaneous platinum needle electrodes placed at the vertex, mastoid prominence, and leg. Response signals were amplified (100,000x), filtered, and acquired by TDT RZ6 (Tucker-Davis Technologies). Each averaged response was based on 1,024 stimulus repetitions. The ABR threshold was defined visually as the lowest sound pressure level at which any wave of the four waves (wave I to wave IV) was detected above the noise level at each frequency of the tone.

Two EC1 electrostatic speakers (Tucker-Davis Technologies) were used for the measurement of DPOAE threshold. Two tone bursts with different frequencies (f_1_ and f_2_, with f_2_/f_1_ = 1.2 and the f_2_ level 10 dB lower than the f_1_ level) were delivered to the ear canal from the speakers through a coupler. The sound pressure obtained from the microphone in the ear-canal was amplified and computed from averaged waveforms of ear-canal sound pressure using the Fast-Fourier transforms. The DPOAE component at the frequency of 2f_1_-f_2_ was measured in response to the two-tone bursts. The DPOAE threshold is defined as the f_1_ sound pressure level (measured in decibels) required to produce a repeatable response above the noise level at the frequency of 2f_1_-f_2_ (16).

### Recording of EP

Details for recording EP are described elsewhere (17, 18). In brief, tracheotomy was performed in the ventral position after anesthesia. The tympanic bulla was opened after tissue and musculature overlying the bulla were removed. A glass capillary pipette electrode (10 MΩ) filled with 3 mM KCl was mounted on a hydraulic micromanipulator. The tip of the pipette electrode was in contact with the round window. With the help of micromanipulator, the electrode was advanced through the round window membrane toward the organ of Corti. After the tip of the microelectrode penetrated through the window membrane and was in the scala tympani, the baseline was adjusted to zero. The microelectrode was then advanced through the region of the organ of Corti on the basilar membrane. When the microelectrode passed through the organ of Corti, a negative DC potential was recorded. A stable positive DC potential (i.e., the EP) was observed when the micropipette entered the scala media. Axopatch 200B amplifier (Molecular Devices, San Jose, CA, USA) was used to record EP. The EP response was amplified under current-clamp mode and acquired by software pClamp 9.2 (Molecular Devices). The sampling frequency was 1 kHz.

### Immunostaining of cochlear and vestibular hair cells and hair cell count

After measuring auditory function, mice were euthanized through CO2 inhalation, and then decapitated to remove the temporal bones. Under a dissecting microscope, a hole was poked at the apex of the cochlea. The round and oval windows were opened with a needle. The temporal bones were fixed in 4% paraformaldehyde in PBS for 2h. After rinsing three times with PBS, the inner ear, containing both the cochlea and vestibular end organs (utricle, saccule and crista), was decalcified in 10% EDTA solution for 1.5 h. The bony wall was then removed and the organ of Corti and vestibular end organs were all dissected out. The auditory and vestibular sensory epithelia were treated with 0.3% Triton X-100 (Sigma-Aldrich, St. Louis, MO, USA) and 5% normal goat serum (ZSGB-BIO, Beijing, China) in PBS for 2h at room temperature. The samples were then incubated at 4°C over-night with the anti-MYO7A antibody (diluted 1:300, Proteus BioSciences Inc., Ramona, CA, USA). After rinsing in PBS, the samples were incubated with secondary antibody tagged with Alexa Fluor 488 (diluted 1:300; Invitrogen, Carlsbad, CA, USA) for 2h at room temperature. Alexa Fluor 594-conjugated phalloidin (diluted 1:300; Invitrogen) was used for labeling F-actin. After rinsing with PBS, samples were mounted on glass slides with Fluoromount-G (Southern Biotech, Birmingham, AL, USA) and examined using a Leica scanning confocal microscope (TCS SP8 II; Leica Microsystems, Wetzlar, Germany). ImageJ (https://imagej.nih.gov/ij/) was used for imaging analysis.

For cochlear hair cell count, images from apical, mid and basal turn regions (each with 400 μm in length) were captured, and IHCs and OHCs were counted separately from confocal images off-line, as described previously (19). For counting hair cells in utricle and saccule from captured confocal images off-line, protocols described previously were used (20). Three cochleae and three utricles and saccules from three animals were used for cell count.

### Semicircular canal histology

The temporal bone tissues were placed in cold 4% paraformaldehyde solution for 24 h, and then in 10% EDTA solution for 3 days. After dehydration in graded concentrations of ethanol, the sample was embedded in graded concentrations of celloidin. Serial sections of the temporal bones were individually cut along the long axis of the two semicircular canals (PSC and LSC) at a thickness of 20 μm. The sections were transferred from an 80% ethanol storage solution and then stained with H and E.

### Scanning electron microscopy

The cochleae from the surgical ear were fixed for 24 h in a solution of 2.5% glutaraldehyde and 0.1 M sodium cacodylate buffer (pH 7.4) containing 2 mM CaCl_2_. The cochlear wall was removed upon decalcification in 10% EDTA solution for 24 h. The cochleae were then post-fixed for 1 h with 1% OsO_4_ in 0.1 M sodium cacodylate buffer and washed. The cochleae were dehydrated *via* an ethanol series, critical point dried from CO_2_ and sputter-coated with gold. The morphology of the HCs was examined in a FEI Quanta 200 scanning electron microscope (ThermoFisher, Hillsboro, OR, USA) and photographed.

### Statistical analyses

Data are expressed as means ± standard errors. Student's *t*-test was used to determine statistical significance between two conditions (control and surgical ears or right and left ears) in hair cell counts or EP change. Two-way ANOVA with multiple *t-*tests using the Holm–Sidak correction for multiple comparisons was also used to determine statistical significance. *P* ≤ 0.05 was regarded as significant.

## Results

**Figure 1:**
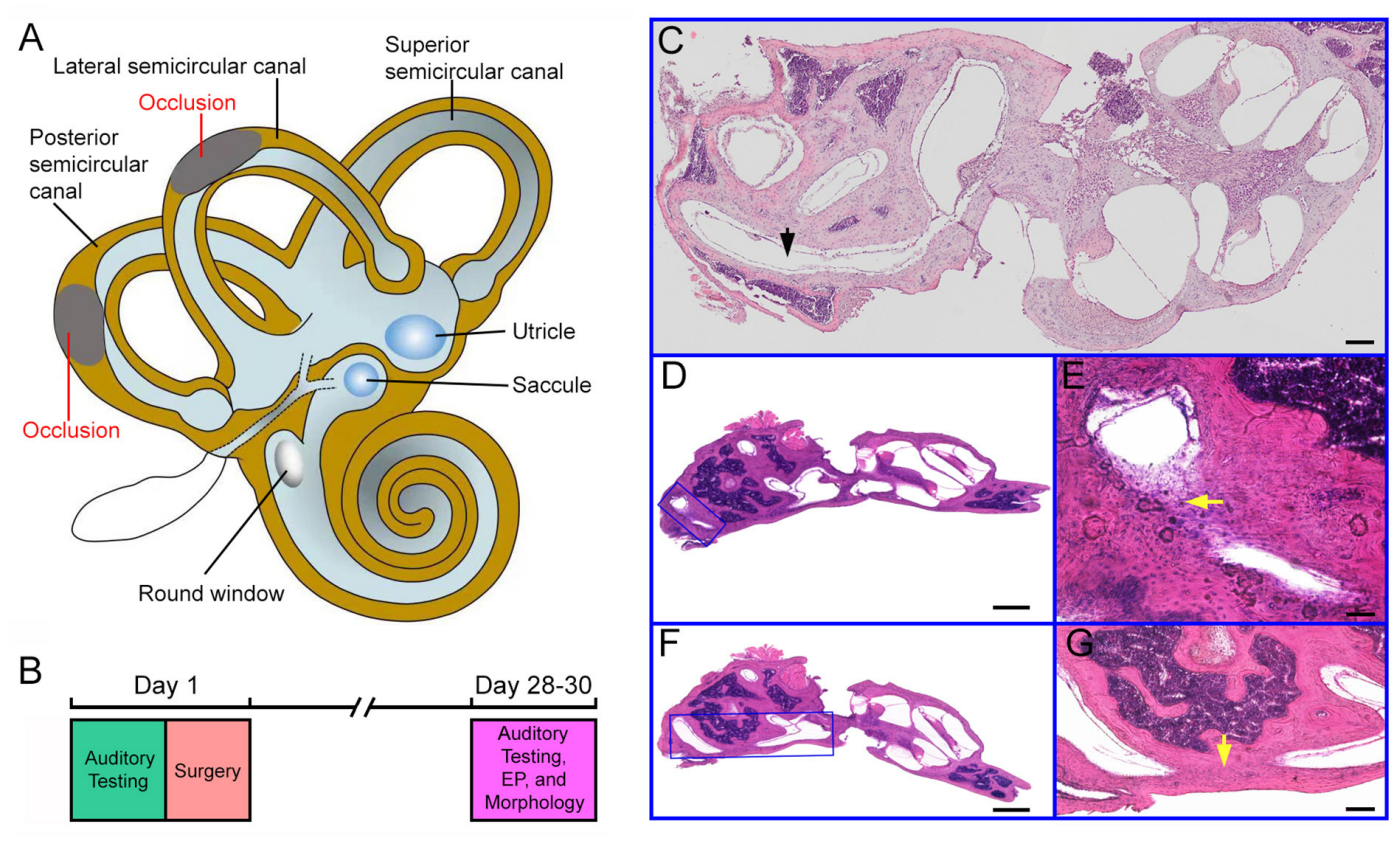
Experimental design and histology of the cochlea and semicircular canals. **(A)** Schematic drawing of the inner ear with the cochlea, the vestibule and the semicircular canals. Occlusions of the lateral and posterior semicircular canals are indicated in the drawing. **(B)** Experimental design and timeline for the surgery and assessment of auditory function and hair cell morphology. **(C)** Cross section (H and E staining) of the inner ear from a control ear. The lateral semicircular canal is marked in the picture. Bar: 50 μm. **(D)** Cross section of the inner ear from a surgical ear with posterior semicircular canal blocked. The area with occlusion is marked with a blue frame. **(E)** High magnification view of the area shown in **(D)**. The area with occlusion is marked by a yellow arrow. **(F)** Image of cross section of the inner ear from a surgical ear with lateral semicircular canal blocked. The area with occlusion is marked with a blue frame. **(G)** High magnification view of the area shown in **(F)**. The area with occlusion is marked by a yellow arrow. Bar for **(D,F)**: 200 μm. Bar for **(E,G)**: 50 μm.

Due to the inaccessibility to the superior semicircular canal during surgery in mice, LSC and PSC were chosen to be blocked in our study (Figure 1A). Tone-evoked ABR thresholds of the mice's bilateral ears were determined before the surgery (designated as Day 1 in Figure 1B) and 4 weeks after the surgery. Four weeks after the surgery ABR and EP were measured and the inner ear morphology was examined, as the experimental design is outlined in Figure 1B.

To determine if the surgery successfully blocked the LSC and PSC, we examined LSC and PSC morphology in celloidin-embedded serial sections. For comparison, morphology of the control ear was also examined. Figures 1C–G shows some representative images of H and E staining of the semicircular canals from the two ears. Figure 1C shows a section of the cochlea and vestibule from a control ear. As shown, the PSC is continuous with no tissue blocking the canal. Figures 1D,F show sections of the surgical ear with LSCO and PSCO at 4 weeks after the surgery. At higher magnification, fibrinous and cellular material was seen in the LSC (Figure 1E) and PSC (Figure 1G) lumen at the surgical site. We examined the morphology of LSC and PSC of the surgical ears in all mice and only those whose LSC and PSC were both blocked were included for analysis.

### Cochlear and vestibular hair cell morphology

**Figure 2:**
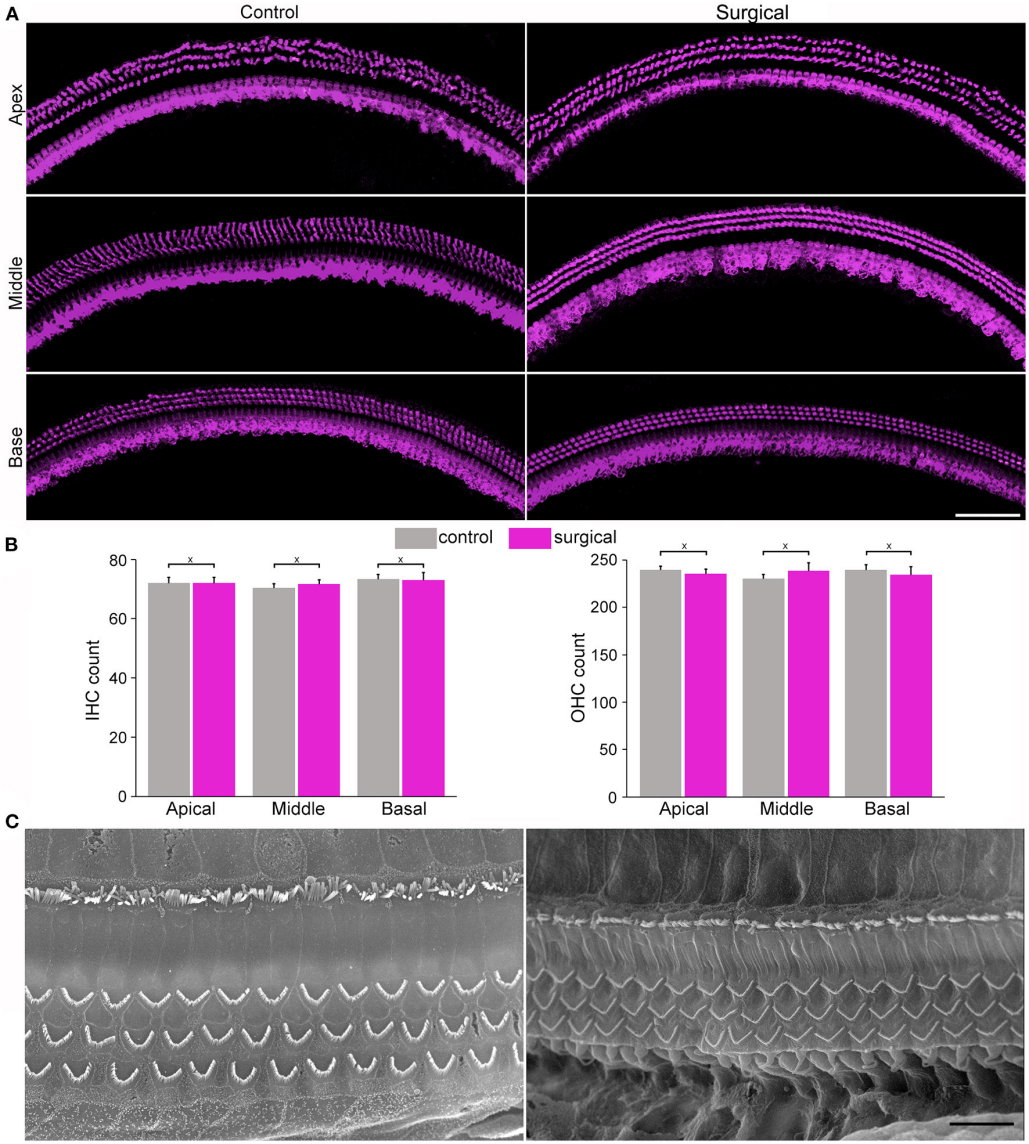
Images from confocal and scanning electron microscopy and morphology of cochlear hair cells and stereocilia. **(A)** Representative confocal images of hair cells from apical, mid- and basal regions of the cochleae from control and surgical ears with occlusion 28 days after surgery (surgical ear was used in all subsequent figures to represent occlusion of two semicircular canals). Bar: 75 μm. **(B)** IHC and OHC count from control and surgical ears. Data are presented as the mean ± SD, *n* = 3. X represents no statistical significance between the two groups (*p* = 0.83, 0.35 and 0.86 for apical, middle and basal turn for IHC count, respectively. *p* = 0.34, 0.22, and 0.44 for apical, middle and basal OHC count, respectively). **(C)** Representative SEM micrographs of stereocilia bundles of hair cells from apical and basal turns of the surgical ear. Bar: 10 μm.

Confocal microscopy was used to examine hair cell status in the cochlea 4 weeks after surgery. Figure 2A show confocal images of hair cells in the three cochlear locations (apical, middle, and basal turn regions) from the surgical and contralateral ears. One row of inner hair cells (IHCs) and three rows of outer hair cells (OHCs) can be seen with no obvious signs of missing hair cells. The number of hair cells in the three cochlear locations from surgical and contralateral ears was counted and the mean of IHC and OHC count is presented in Figure 2B, respectively. We compared the hair cell count between surgical and contralateral ear at these locations and no statistical difference was found (*n* = 3, *P* > 0.05). We also used scanning electron microscopy to examine stereocilia morphology of hair cells in the surgical ear. Figure 2C shows two representative SEM micrographs obtained from the apical and basal turn regions. The stereocilia appear to be normal with no signs of degeneration, such as fusion and loss.

**Figure 3:**
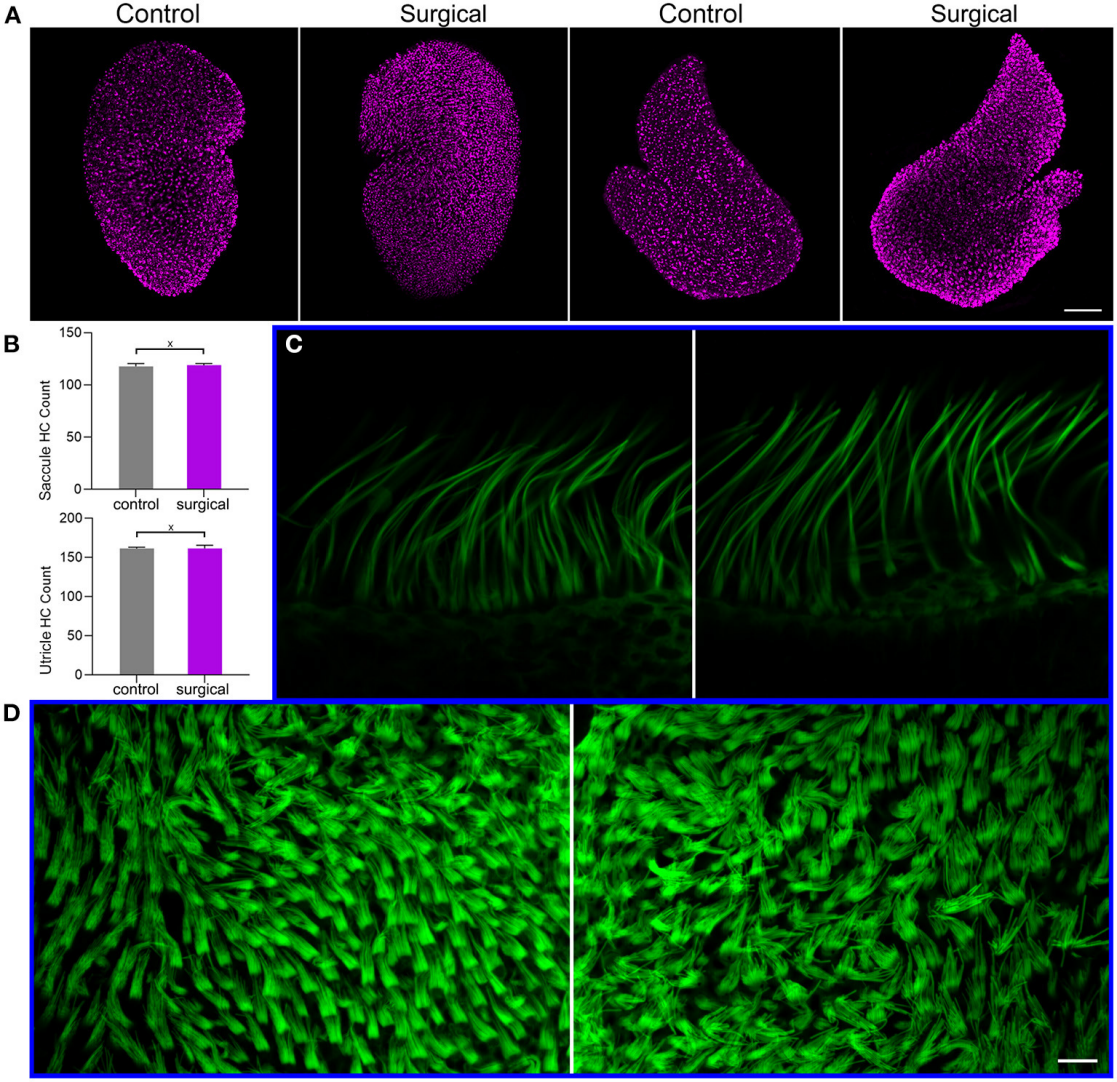
Hair cell and stereocilia status after occlusion of lateral-posterior semicircular canals. **(A)** Confocal images of utricle and saccule hair cells in control and surgical ears. Hair cells were labeled with anti-MYO7A antibody. Bar: 75 μm. **(B)** Hair cell count in utricle and saccule of control and surgical ears. X marks no statistical significance between control and surgical ears (*p* = 0.35 and 0.48 for saccule and utricle hair cell count, respectively). Data are presented as the mean ± SD, *n* = 3. **(C)** Confocal images of stereocilia bundles of crista hair cells from control (left panel) and surgical (right panel) ears. **(D)** Confocal images of utricle hair cell stereocilia control (left panel) and surgical (right panel) ears. Bar: 10 μm for **(C,D)**.

Vestibular hair cell morphology was examined in the surgical ear to determine if blockage of the lateral and posterior canals causes degeneration of hair cells in utricle, saccule and crista. Confocal microscopy was used to examine hair cell morphology. Figure 3A shows confocal images of anti-MYO7A antibody-labeled hair cells in utricle and saccule of surgical and contralateral (control) ears. The number of hair cells in utricle and saccule was counted and compared between surgical and control ears. The mean count is presented in Figure 3B. No statistical significance was found between control and surgical ears. We did not count the hair cell number in crista as it is not easy to accurately count hair cells in crista in the surface mount (whole mount) preparation. But we examined stereocilia bundle morphology of hair cells in crista (Figure 3C) and utricle (Figure 3D) in the control and surgical ears. As shown, the stereocilia bundles appear to be normal with no signs of loss and degeneration.

### ABR and DPOAE thresholds

**Figure 4:**
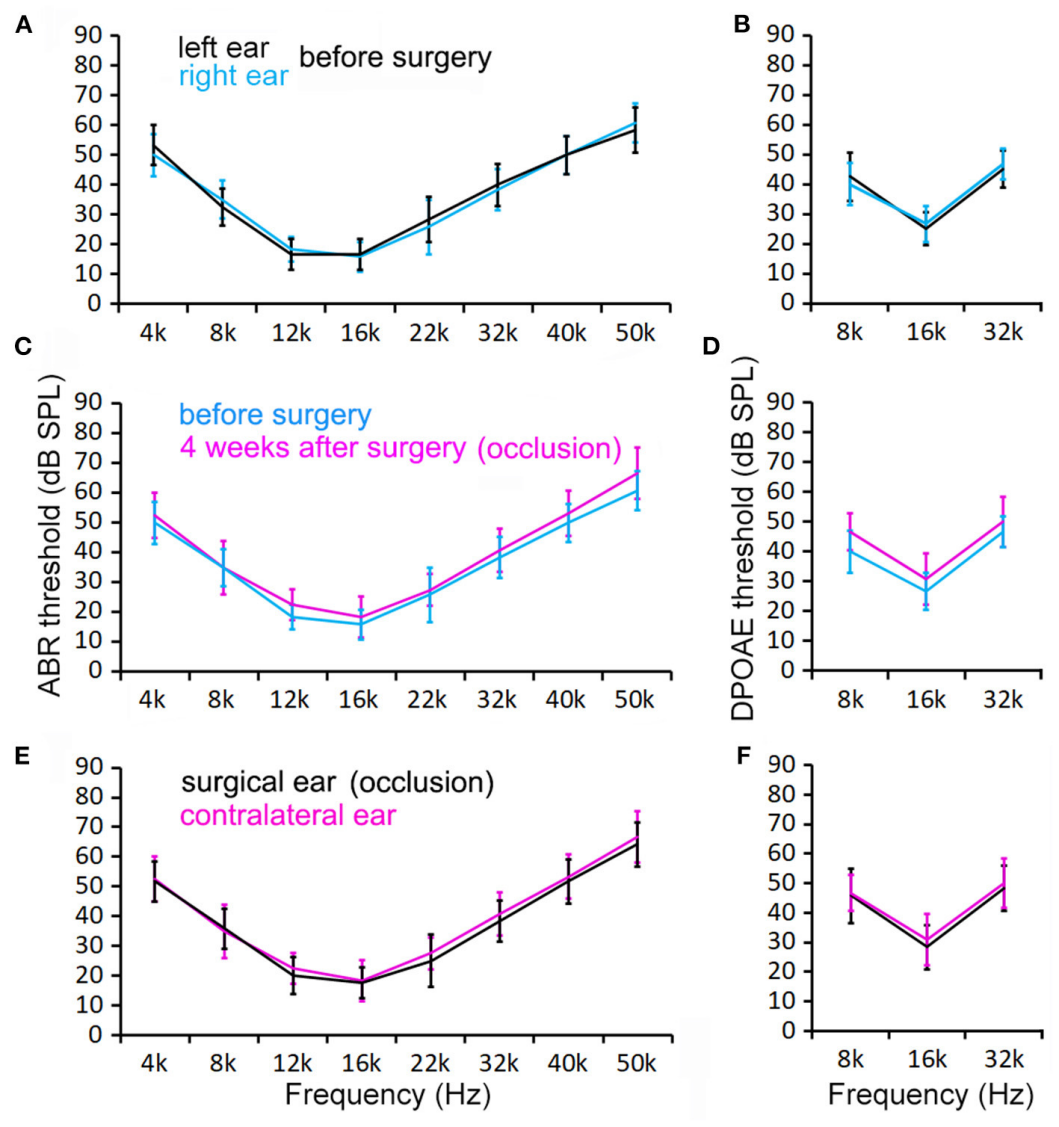
ABR and DPOAE thresholds measured from control and surgical ears before and 4 weeks after surgery. **(A,B)** ABR and DPOAE thresholds of the right and left ears before surgery. No statistical significance in thresholds is found at any of these frequencies (*p* > 0.05, *n* = 6). **(C,D)** ABR and DPOAE thresholds of the ears before and after occlusion. No statistical significance in thresholds is found at any of these frequencies (*p* > 0.05, *n* = 6). **(E,F)** Control and surgical ears at 4 weeks after surgery. No statistical significance in thresholds is found at any of these frequencies (*p* > 0.05, *n* = 6). Two-way ANOVA with multiple *t-*tests using the Holm–Sidak correction for multiple comparisons was also used to determine statistical significance.

To determine if SCO leads to hearing loss, we measured ABR and DPOAE thresholds. Figures 4A,B show the mean ABR and DPOAE thresholds measured from the left and right ears from six mice before the surgery. The ABR and DPOAE thresholds (at each frequency) of the two ears were not observed to be not statistically different (*P* > 0.05 for ABR and DPOAE, *n* = 6). Figures 4C,D show the mean ABR and DPOAE thresholds measured from the surgical ear before and 4 weeks after the surgery. No significant difference in either ABR or DPOAE threshold was found (*P* > 0.05 for ABR and DPOAE, respectively, *n* = 6). We also compared ABR and DPOAE thresholds between the surgical and contralateral ears after surgery, in order to minimize the possible effect of age-related hearing loss on our measurement. As shown in Figures 4E,F, no significant difference in either ABR or DPOAE threshold is found (*p* > 0.05 for ABR and DPOAE, respectively, *n* = 6).

### EP measured from surgical ear

**Figure 5:**
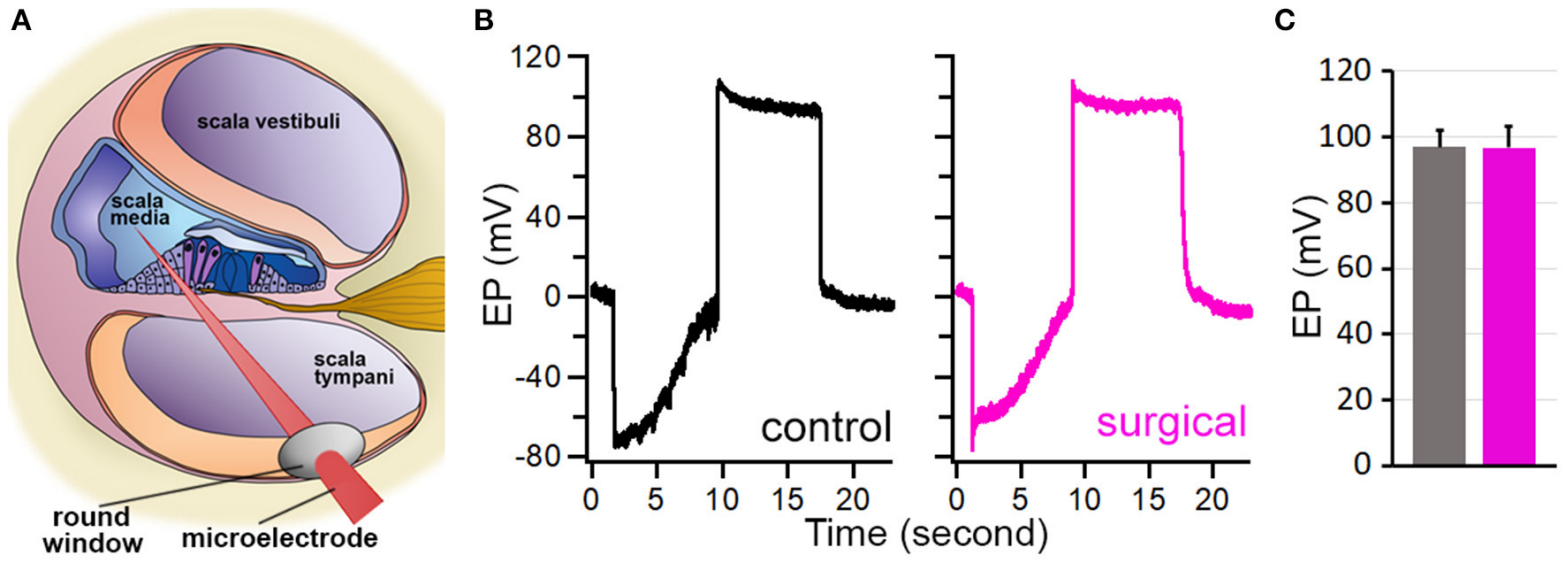
EP measured from control and surgical ears. **(A)** Schematic drawing of EP measurement. **(B)** Representative EP measured from control and surgical ears. **(C)** Mean ± SD of EPs measured from control and surgical ears. No statistical difference was found (*p* = 0.72, *n* = 6).

The endolymph within the scala media exhibits a constant ~80-mV positive polarization with respect to the perilymph (21). This positive potential or EP provides a driving force for hair cell mechanotransduction. We measured EP to determine if SCO would affect EP magnitude as endolymph dynamic in the endolymph compartment might have been altered by blockage of the two semicircular canals. We took the round window approach to measure EP (17, 18), as shown in Figure 5A. Figure 5B exhibits two examples of EP measured from the surgical and contralateral ears 4 weeks after the surgery. The negative potential shown in the figure reflects the organ of Corti potential, while the positive potential is the EP (22). As displayed, the EP magnitude is similar between the two ears. We measured EP from the surgical and contralateral ears from six mice and the mean EP magnitude is presented in Figure 5C. The EP magnitude is 98.3 ± 6.3 mV for the surgical ear and 96.7 ± 5.6 mV for the contralateral ear. No statistical significance (*p* = 0.71, *n* = 6) was found between the two ears after the surgery.

## Discussion

It has been known for a long time that surgically obstructing posterior semicircular canal is an effect way to treat benign positional vertigo (8–10, 13, 23). The main risk of such an operation was the potential damage to hearing. The first report of a successful posterior canal occlusion for posterior canal benign positional vertigo was in two patients with profound deafness (8), and a year later in five patients with normal hearing (7). In a different study, 53 patients underwent posterior canal occlusion and all 53 were cured of their benign positional vertigo. Nine suffered some symptomatic permanent hearing loss between 20 to 25 dB at low to high frequencies (10). Experimental semicircular canal plugging has long been performed in animal models for research purposes (24–29). Plugging individual semicircular canals reduces only the activity of affected crista without influencing the function of the remaining vestibular labyrinth in cats (26) and squirrel monkeys (30). Parnes and McClure (27) examined the auditory function of guinea pigs by measuring ABR before and after plugging posterior semicircular canal. They found ABR thresholds were not affected. However, the results are not entirely consistent since ABR thresholds were reported to be elevated in some human and animal studies (7, 9, 24). The exact reason of why hearing was affected in some animals and human patients was unknown (31), however, damage during surgery and/or different ways to plug the canal may have played a role (24, 31).

In the present study we show that ABR and DPOAE thresholds measured from the ear with occlusion of lateral and posterior semicircular canals were not significantly elevated when compared to the pre-operative thresholds and thresholds of the contralateral ear. Although previous studies also examined auditory function after SCO, our study differs from previous studies. First, we examined auditory function after two canals (PSC and LSC) were plugged. In most previous studies, only one canal, often PSC, was plugged. Second, we measured EP to determine if occlusion of two canals would affect EP. Although one study also measured EP after three canals were blocked in a guinea pig model, the EP was only monitored for a short period of time right after the surgery (25). No long-term effect of canal obstruction was examined. Third, our study is more comprehensive; we not only measured ABR and DPOAE thresholds and examined hair cell morphology and stereocilia ultrastructure, but also measured EP. Most previous studies only measured ABR threshold and/or examined cochlear hair cell morphology.

Transient loss of hearing and vestibular function has been seen in patients and animal models after SCO procedures (7, 9, 10, 32). We did not examine auditory function in the first few days after the surgery as our focus was on the long-term effect. However, we observed circular behavior in some mice, suggesting a transient effect on the vestibular function. The circular behavior disappeared in 3 to 5 days after surgery in mice used in our study, suggesting compensation and adaption to angular acceleration in two ears. We note that the absence of circular behavior does not necessarily mean that the response to rotational movements is not affected, as the goal of occlusion of semicircular canals is to reduce stimulation to crista hair cells to treat intractable vertigo symptoms.

There are two limitations to our study. The first limitation is that our conclusion that SCO exhibited no obvious effect on auditory function was only based on observations made in a period of 4 weeks after the surgery. Therefore, it is unclear if the procedure would have any negative impact on long-term auditory function. As obstruction of the canals is certain to lead to change in endolymph flow, it is yet to be determined if such change could eventually lead to endolymphatic hydrops (29). The second limitation is that we did not examine vestibular function after SCO surgery in our animal models. Permanent impairment of semicircular canal function was observed in the cat (26), guinea pig (33) and human (34). In guinea pigs, vestibulo-ocular reflex to high acceleration impulsive head rotations was lost following a unilateral lateral semicircular canal occlusion and no adaptive plasticity was found (33). Interestingly, in clinical studies the majority of patients who received posterior canal occlusion surgery did not show signs of loss of balance (10). However, incomplete occlusion or ossification of SCs was the principal cause of vertigo recurrence in Meniere's disease patients who underwent triple semicircular canal plugging (32).

In summary, our results revealed that the plugging two semicircular canals does not lead to detectable hearing loss. Hair cell morphology in the cochlea and vestibule appears to be normal with no signs of hair cell degeneration and loss. EP also shows no significant change despite change in the endolymph fluid movement in the canal. The fact that normal hearing is preserved in the mouse model with obstruction of two canals suggest that SCO procedure does not disrupt auditory function. The procedure can be extended to human patients with normal hearing to treat benign positional vertigo after vestibular function and balance of the patient are also considered in decision making about SCO surgery.

## Data availability statement

The raw data supporting the conclusions of this article will be made available by the authors, without undue reservation.

## Ethics statement

The animal study was reviewed and approved by Animal Care Committee of Beijing Tongren Hospital.

## Author contributions

TW performed the experiments, analyzed the data, and wrote the manuscript. HL and DH performed some experiments. YL and DH designed the experiments, analyzed the data, and revised and finalized the manuscript. All authors contributed to the article and approved the submitted version.

## Funding

This work was supported by National Natural Science Foundation of China grants #81870718 and #81770996 to YL.

## Conflict of interest

The authors declare that the research was conducted in the absence of any commercial or financial relationships that could be construed as a potential conflict of interest.

## Publisher's note

All claims expressed in this article are solely those of the authors and do not necessarily represent those of their affiliated organizations, or those of the publisher, the editors and the reviewers. Any product that may be evaluated in this article, or claim that may be made by its manufacturer, is not guaranteed or endorsed by the publisher.
